# Absent in melanoma 2 suppresses gastric cancer cell proliferation and migration via inactivation of AKT signaling pathway

**DOI:** 10.1038/s41598-021-87744-4

**Published:** 2021-04-15

**Authors:** Dong Wang, Junwei Zou, Jun Dai, Zhengwu Cheng

**Affiliations:** 1 Department of Hepatobiliary Surgery, The First Affiliated Hospital of Wannan Medical College, Wuhu, 241000 China; 2 Department of General Surgery, The Second Affiliated Hospital of Wannan Medical College, Wuhu, 241000 China; 3 Department of Gastrointestinal Surgery, The First Affiliated Hospital of Wannan Medical College, No.2 Zheshan West Road, Jinghu District, Wuhu, 241000 Anhui Provinc China

**Keywords:** Cancer, Gastric cancer

## Abstract

Gastric cancer (GC) is the third leading cause of cancer-related mortality worldwide, and poses a great threat to public health. Absent in melanoma 2 (AIM2), a member of the pyrin-HIN family proteins, plays various roles across different types of cancers. However, the possible role of AIM2 in GC, as well as the underling mechanisms, are equivocal and need to be further explored. Herein, we identified that AIM2 expression was significantly down-regulated in GC tissues. Furthermore, loss of AIM2 was significantly associated with tumor size, lymph node metastasis (LNM) and tumor, node, metastases (TNM) staging, as well as poor prognosis in GC patients. Knockdown of AIM2 in GC cells significantly promoted cellular proliferation and migration, whereas AIM2 overexpression did the opposite. Mechanistically, we discovered that AIM2 regulates the AKT signaling pathway. In fact, the enhanced proliferation and migration ability induced by AIM2 knockdown was partially impaired in cells treated with the AKT inhibitor. Overall, our findings suggests that AIM2 is an independent prognostic marker and highlights a new entry point for targeting the AIM2/AKT signaling axis for GC treatment.

## Introduction

Gastric cancer (GC) represents the fifth highest incidence among all human malignancies, with over a million new cases being diagnosed globally every year^1,2^. GC is also the third leading cause of cancer-related death worldwide, and poses a great threat to public health^3,4^. Unfortunately, a majority of GC patients are diagnosed at an advanced stage, thus missing the best timing for radical surgery and resulting in poor prognosis^3,4^. Therefore, there is an urgent need to identify novel biomarkers and therapeutic targets for patients with GC.

Absent in melanoma 2 (AIM2), a member of the interferon-inducible PYRIN and HIN domain-containing (PYHIN) family of proteins, binds to cytosolic double-stranded DNA (dsDNA) and recruits the adapter ASC (apoptosis-associated speck-like protein containing a CARD) in order to promote caspase-1 activation. Subsequently, activated caspase-1 cleaves the proinflammatory IL-1β and IL-18 into their bioactive forms, causing a type of inflammatory cell death. Thus, AIM2 plays a role in the host defense against bacterial and viral pathogens^5–9^.

In addition to having a function in innate immunity, AIM2 is also known to be involved in the development and progression of cancers. Prior studies have indicated that AIM2 functions as both a tumor suppressor and oncogene across different types of cancers. In a breast cancer model, AIM2 is reported to suppress cellular proliferation in vitro and mammary tumor growth within a mouse model^10^. Furthermore, AIM2 expression is decreased in hepatocellular carcinoma, and low AIM2 expression contributes to hepatocarcinoma tumorigenesis and metastasis^11,12^. Moreover, several studies have demonstrated that loss of AIM2 expression exhibits oncogenic properties in colorectal cancer and low levels of AIM2 predict poor survival in colorectal cancer patients^13–16^. On the other hand, recent studies have also demonstrated that AIM2 is highly expressed in oral squamous cell carcinoma^17^ and non-small cell lung cancer^18,19^. Additionally, knockdown of AIM2 in these cells leads to an inhibition of tumor cell growth and migration, suggesting that AIM2 functions as an oncogene^17–19^. In GC, AIM2 expression is reported to be significantly lower in early GC tissues compared to progressive GC tissues, and AIM2 overexpression exerts a suppressive effects on GC cell proliferation, migration and invasion^20^. However, the clinical significance and underling mechanisms of AIM2 in GC have not yet been elucidated and need to be further explored.

Herein, we report that AIM2 expression is significantly down-regulated in GC tissues, and that reduced AIM2 expression predicts poor prognosis of GC patients. We also demonstrate a suppressive role of AIM2 in GC cell proliferation and migration. Moreover, our study demonstrates that AIM2 inhibits phosphorylation and activation of AKT, which may be responsible for the effect of AIM2 on GC cell proliferation and migration. Therefore, our data suggests that AIM2 is a novel tumor suppressor, and highlights the possible role of AIM2 as a therapeutic target for GC treatment.

## Results

### AIM2 expression in human GC tissues

**Figure 1:**
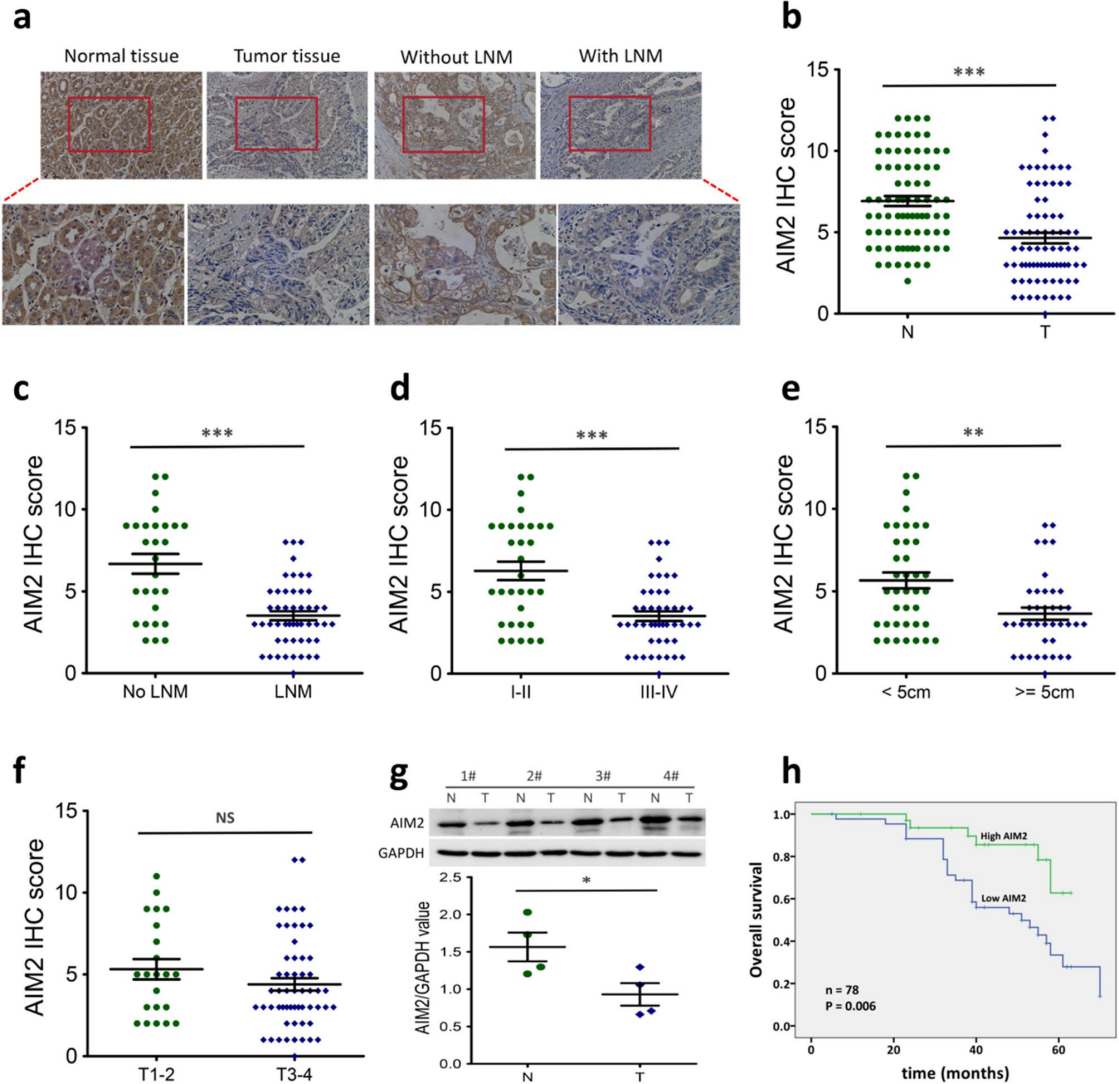
AIM2 is down-regulated in clinical GC tissues. **(a**) IHC staining of AIM2 in representative carcinoma and the surrounding tissues of GC. (**b**) Scatter plot analysis of AIM2 levels in 78 cases of GC tissue samples (T) and matched normal tissues (N). (**c**) Scatter plot analysis of AIM2 levels in GC tissues with (n = 50) or without LNM (n = 28). (**d**) Scatter plot analysis of AIM2 levels in GC tissues with TNM stage I–II (n = 32) or stage III–IV (n = 46). (**e**) Scatter plot analysis of AIM2 levels in GC tissues with tumor size < 5 cm (n = 39) or ≥ 5 cm (n = 39). (**f**) Scatter plot analysis of AIM2 levels in GC tissues with depth of invasion T1–2 (n = 22) or T3–4 (n = 56). (**g**) Immunoblotting for AIM2 protein in four randomly selected pairs of GC tumors (T) and their surrounding tissues (N) are presented (top). GAPDH as a loading control. Scatter plot analysis of AIM2/GAPDH values in GC tissues (bottom) The proteins were quantified using the ImageJ software (version: 1.4.3). (**h**) Kaplan–Meier survival curve of GC patients with low or high AIM2 expression. NS, nonsignificant; *P < 0.05; **P < 0.01; ***P < 0.001. The original Western blots are included in the Supplementary Fig. S1.

In order to investigate the possible function of AIM2 in GC, we initially analyzed AIM2 expression in GC tissues. Immunohistochemical staining indicated that AIM2 is down-regulated in GC tumor tissues compared to normal tissues (*P* < 0.001; Fig. 1a,b). Statistically, AIM2 was found to be low-expressed in 56.4% of GC cases (n = 44); however, only 23.1% of cases (n = 18) exhibited negative or low expression among the non-tumorous tissues (Table 1). Further analysis indicated that AIM2 levels were lower in tumor tissues with lymph node metastasis (LNM) than those without LNM (*P* < 0.001; Fig. 1a,c). Additionally, AIM2 expression was further reduced in GC tissues with tumor-node-metastasis (TNM) stage III–IV (*P* < 0.001) and with tumor size ≥ 5 cm (*P* < 0.01), compared to TNM stage I–II and tumor size < 5 cm, respectively (Fig. 1d,e). On the other hand, we found similar AIM2 expression across samples, regardless of different depth of invasion (T1–2 vs. T3–4; *P* > 0.05; Fig. 1F). Next, we conducted Western blot analysis to assess the protein expression of AIM2 across four pairs of GC tumor tissues, and adjacent non-tumorous tissues. In accordance with the IHC analyses, results from Western blots confirmed that AIM2 expression is significantly lower in GC tumor tissues compared to paired normal tissues (*P* < 0.05, Fig. 1g).

**Table 1:** The association of AIM2 expression with clinicopathological features in 78 gastric cancers.

Factors	Total cases	AIM2 expression		χ^2^	*P* value
		None or low	High		
**No. of patients**	78	44 (56.4%)	34 (43.6%)		
**Age**					
< 65	41	22 (53.7%)	19 (46.3%)	0.266	0.606
≥ 65	37	22 (59.5%)	15 (40.5%)		
**Sex**					
Male	51	29 (56.9%)	22 (43.1%)	0.012	0.912
Female	27	15 (55.6%)	12 (44.4%)		
**Tumor size**					
< 5 cm	39	16 (41.0%)	23 (59.0%)	7.508	0.006**
≥ 5 cm	39	28 (71.8%)	11 (28.2%)		
**Tumor location**					
U	43	25 (58.1%)	18 (41.9%)	0.117	0.733
L	35	19 (54.3%)	16 (45.7%)		
**Depth of invasion**					
T1–2	22	9 (40.9%)	13 (59.1%)	2.994	0.084
T3–4	56	35 (62.5%)	21 (37.5%)		
**Lymph node metastasis**					
No	28	8 (28.6%)	20 (71.4%)	13.767	< 0.001***
Yes	50	36 (72.0%)	14 (28.0%)		
**TNM stage**					
I/II	32	10 (31.3%)	22 (68.7%)	13.969	< 0.001***
III/IV	46	34 (73.9%)	12 (26.1%)		

**P < 0.01, ***P < 0.001.

Next, we evaluated the clinical relevance of AIM2 in GC. We analyzed the relationship between AIM2 expression and various clinicopathological parameters in GC patients. AIM2 expression was found to be significantly associated with tumor size (*P* = 0.006), LNM (*P* < 0.001) and TNM stage (*P* < 0.001) (Table 1). However, we found no significant correlation between AIM2 expression and other clinicopathological features, including age, gender, tumor location and depth of invasion.

These results reveal that AIM2 is down-regulated in GC tissues and that reduced AIM2 expression is correlated with clinicopathological parameters in GC patients, which suggests that AIM2 plays a role in the development and progression of GC.

### Prognostic significance of AIM2 in GC

Next, we investigated the prognostic role of AIM2 in GC patients. The overall survival (OS) curve of AIM2 was plotted by the AIM2 expression levels, and calculated by the Kaplan–Meier method. Results indicated that GC patients with low AIM2 expression had significantly lower survival rates compared to those with high AIM2 expression (Fig. 1h), which indicates that AIM2 may function as a prognostic marker in GC. Additionally, univariate analysis (Table 2) further revealed that tumor size (HR: 3.870; 95% CI 1.764–8.490; *P* = 0.001), depth of invasion (HR: 5.920; 95% CI 1.410–24.856; *P* = 0.015), LNM (HR: 0.101; 95% CI 0.024–0.423; *P* = 0.002), TNM stage (HR: 0.130; 95% CI 0.040–0.429; *P* = 0.001) and AIM2 expression (HR: 0.135; 95% CI 0.041–0.443; *P* = 0.001) were all statistically significant prognostic factors. Importantly, multivariate analysis demonstrated that low AIM2 expression is an independent prognostic factor for survival of GC patients (HR: 0.218; 95% CI 0.059–0.806; *P* = 0.029).

**Table 2:** Univariate and multivariate analysis of overall survival (Cox’s regression model).

Factors	Univariate analysis			Multivariate analysis		
	HR	95% CI	*P* value	HR	95% CI	*P* value
Age (< 65/≥ 65)	1.378	0.674–2.815	0.380	1.247	0.531–2.930	0.612
Gender (Male/Female)	0.676	0.310–1.473	0.324	0.915	0.378–2.212	0.843
Tumor size (< 5/≥ 5 cm)	3.870	1.764–8.490	0.001**	1.317	0.500–3.470	0.577
Tumor location (U/L)	1.049	0.500–2.200	0.900	0.685	0.320–1.467	0.330
Depth of invasion (T1–2/T3–4)	5.920	1.410–24.856	0.015*	2.752	0.144–52.462	0.501
Lymph node metastasis (no/yes)	0.101	0.024–0.423	0.002**	0.227	0.013–3.982	0.310
TNM stage (I–II/III–IV)	0.130	0.040–0.429	0.001**	1.180	0.034–40.857	0.927
AIM2 expression (low/high)	0.135	0.041–0.443	0.001**	0.218	0.059–0.806	0.022*

*P < 0.05,**P < 0.01. *HR* hazard ratio, *CI* confidence interval.

### AIM2 expression in GC cells

**Figure 2:**
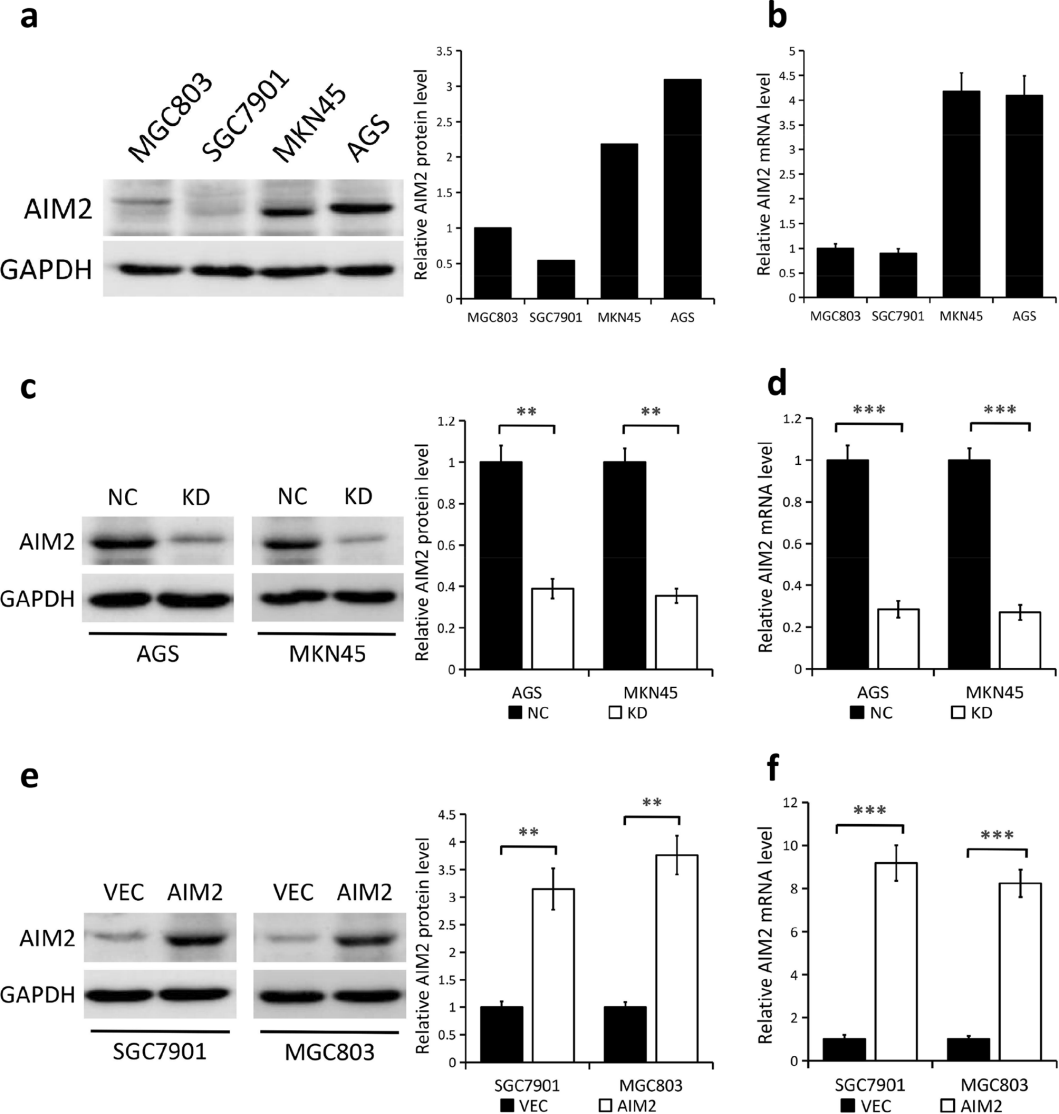
Knockdown and overexpression efficiency of AIM2 in GC cell lines. (**a**,**b**) The protein (**a**) and mRNA (**b**) expression of AIM2 in four human GC cell lines (MGC803, SGC7901, MKN45 and AGS). (**c**,**d**) The protein (**c**) and mRNA (**d**) expression of AIM2 in wild-type AGS and MKN45 cells (NC) and in cells with stable knockdown of AIM2 (KD). (**e**,**f)** The protein (**e**) and mRNA (**f**) expression of AIM2 in wild-type SGC7901 and MGC803 cells (VEC) and in cells with stable overexpression of AIM2 (OE). GAPDH as a loading control. The proteins were quantified using the ImageJ software (version: 1.4.3). **P < 0.01. ***P < 0.001. The original Western blots are included in the Supplementary Fig. S2.

Next, we detected AIM2 protein and mRNA expression levels across four human GC cell lines (MGC803, SGC7901, MKN45 and AGS) using Western blot and q-PCR analyses, respectively. Compared to low levels of AIM2 in the MGC803 and SGC7901 cells, AIM2 expression was found to be much higher in MKN45 and AGS cells, at both the protein and mRNA levels (Fig. 2a,b). Therefore, we utilized a lentiviral shRNA technique in order to achieve a stable knockdown of AIM2 in MKN45 and AGS cells. We also transfected MGC803 and SGC7901 cells with plasmids encoding human *AIM2* to obtain AIM2-overexpression cell lines. AIM2 protein (*P* < 0.01) and mRNA (*P* < 0.001) expression were both significantly reduced in AGS and MKN45 cells with stable knockdown of AIM2 compared to the wild-type cells (Fig. 2c,d). Conversely, the AIM2-overexpression SGC7901 and MGC803 cell lines expressed high level of AIM2 at both the protein (*P* < 0.01, Fig. 2e) and mRNA (*P* < 0.001, Fig. 2f) levels, compared to the control groups.

### Effects of AIM2 on GC cell proliferation and migration

**Figure 3:**
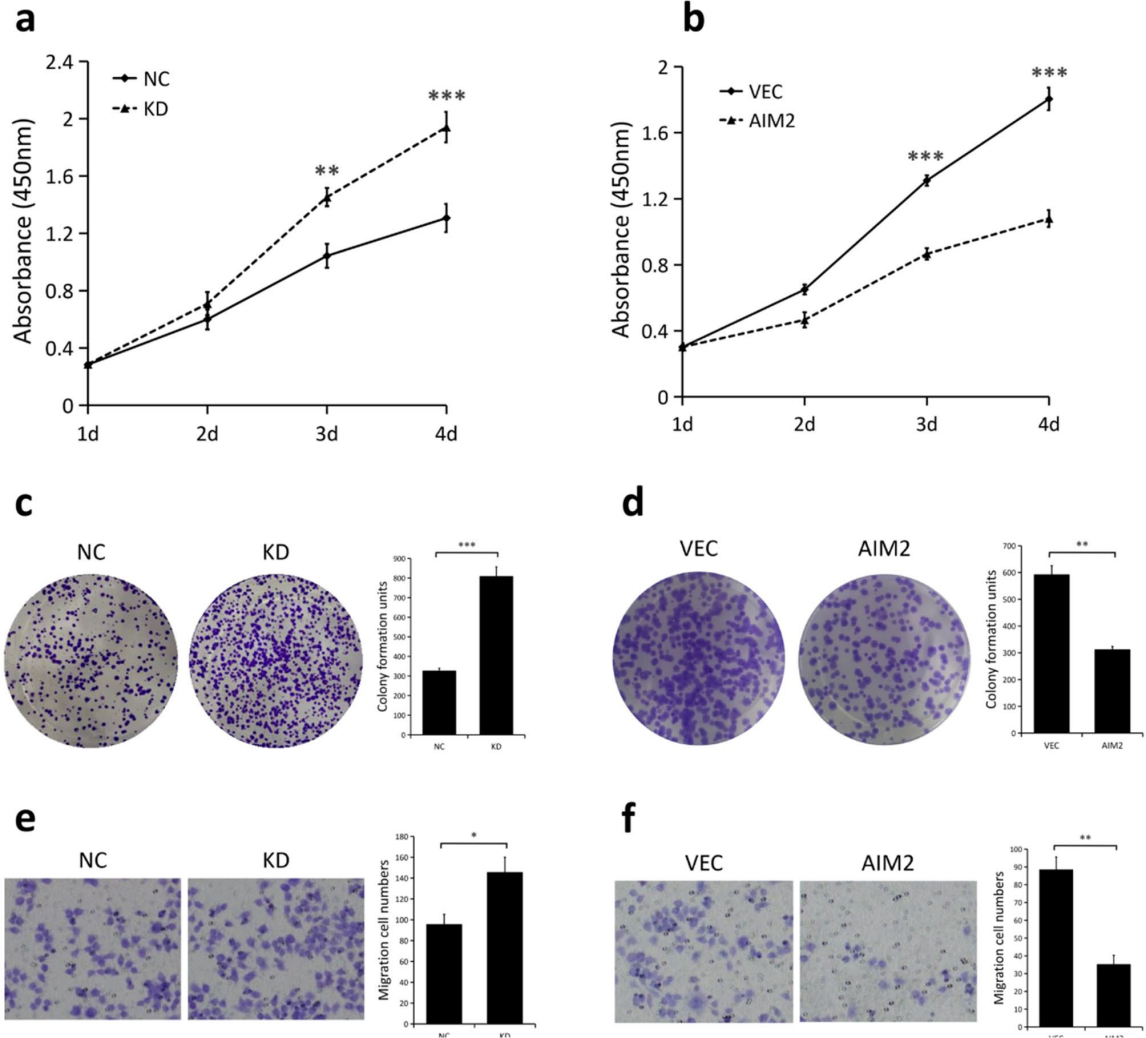
Effects of AIM2 on GC cell proliferation and migration. (**a**) CCK8 assays were performed in wild-type AGS cells (NC) and in cells with stable knockdown of AIM2 (KD). (**b**) CCK8 assays were performed in wild-type SGC7901 cells (VEC) and in cells with stable overexpression of AIM2 (OE). (**c**) Colony formation assays were performed in AGS cells (NC vs. KD) and the relative number of colonies was quantified (right). Error bars, mean ± SEM from three biological replicates. (**d**) Colony formation assays were performed in SGC7901 cells (VEC vs. OE) and the relative number of colonies was quantified (right). Error bars, mean ± SEM from three biological replicates. (**e**) Migration assays were performed in AGS cells (NC vs. KD), and the migratory cells number was quantified (right). Error bars, mean ± SEM from three biological replicates. **f** Migration assays were performed in SGC7901 cells (VEC vs. OE), and the migratory cells number was quantified (right). Error bars, mean ± SEM from three biological replicates. Statistical significance was analyzed by a two-tailed, unpaired Student t-test. *P < 0.05; **P < 0.01; ***P < 0.001.

We performed CCK8 assays in order to determine the effect of AIM2 on GC cell proliferation. Results indicated that AIM2 knockdown significantly enhanced proliferation of AGS cells (*P* < 0.001; Fig. 3a), while AIM2 overexpression inhibited SGC7901 cellular proliferation (*P* < 0.001; Fig. 3b). Consistent with CCK8 results, data from the colony formation assays demonstrated that the ability of AGS cells to form foci was significantly enhanced when cells lacked AIM2 (*P* < 0.001, Fig. 3c), On the other hand, elevated AIM2 expression in SGC7901 cells strikingly impaired cells to develop colonies (*P* < 0.01, Fig. 3d). In a transwell assay, depleted AIM2 expression increased migration of AGS cells (*P* < 0.05, Fig. 3e). On the contrary, SGC7901 cells with AIM2 overexpression demonstrated a diminished ability to migrate (*P* < 0.01, Fig. 3f). Collectively, these data indicate that AIM2 has a suppressive role in GC cell proliferation and migration.

### AIM2 inhibits GC cell proliferation and migration through AKT signaling pathway

**Figure 4:**
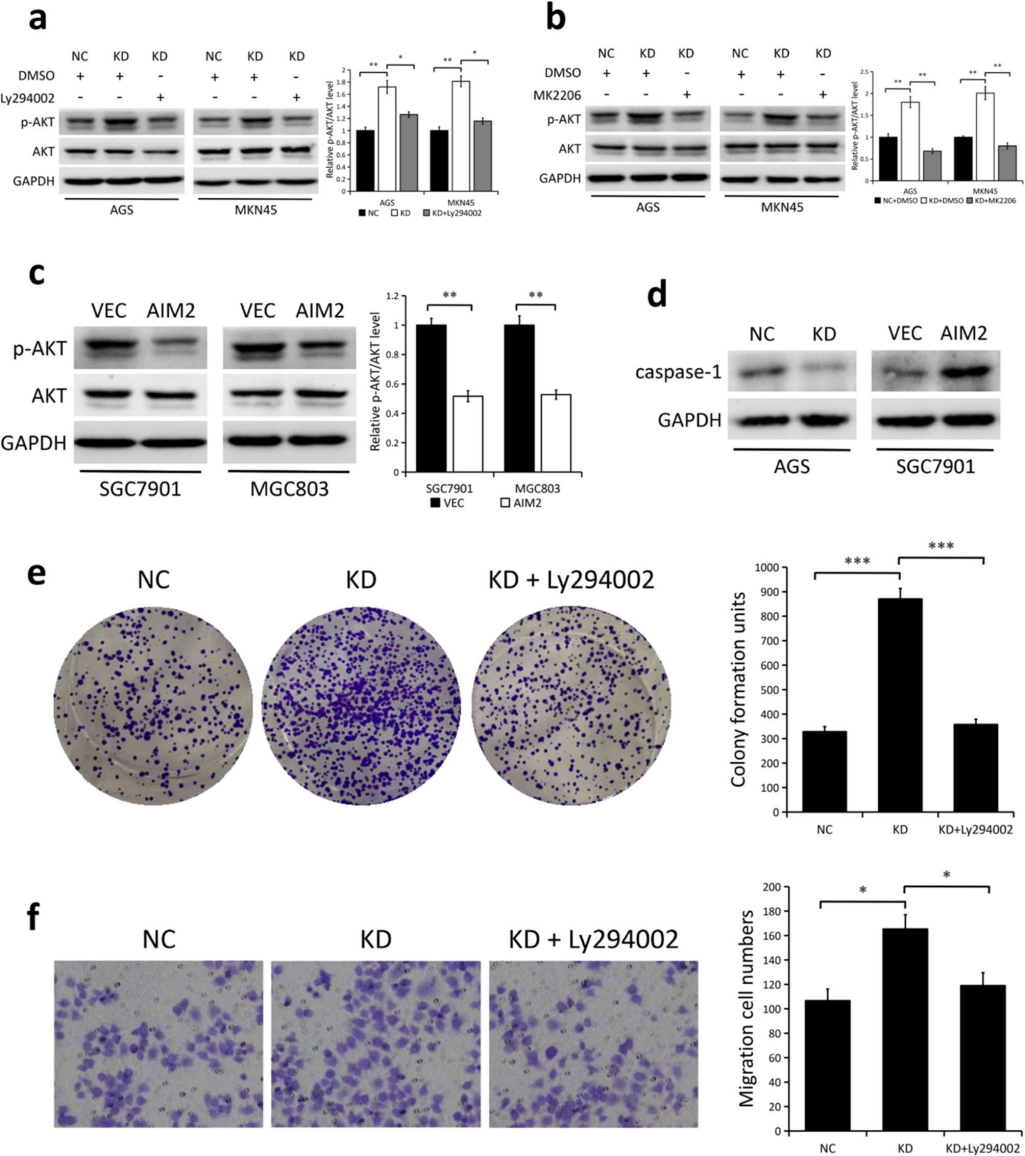
AIM2 inhibits GC cell proliferation and migration in an AKT-dependent manner. (**a**,**b**) Immunoblotting of the indicated proteins in wild-type AGS and MKN45 cells (NC) and in cells with stable knockdown of AIM2 (KD) with or without treatment of Ly294002 (**a**, 20 μM) or MK2206 (**b**, 5 μM). DMSO treatment is considered as the control group. GAPDH as a loading control. (**c**) Immunoblotting of the indicated proteins in wild-type SGC7901 and MGC803 cells (VEC) and in cells with stable overexpression of AIM2 (OE). GAPDH as a loading control. (**d**) Immunoblotting of caspase-1 in AGS cells (NC vs. KD) and SGC7901 cells (VEC vs. AIM2). GAPDH as a loading control. (**e**) Colony formation assays were performed in AGS cells (NC vs. KD) in the presence or absence of Ly294002, and the relative number of colonies was quantified (right). Error bars, mean ± SEM from three biological replicates. (**f**) Migration assays were performed in AGS cells (NC vs. KD) in the presence or absence of Ly294002, and the migratory cells number was quantified (right). Error bars, mean ± SEM from three biological replicates. The proteins were quantified using the ImageJ software (version: 1.4.3). *P < 0.05; **P < 0.01; ***P < 0.001. The original Western blots are included in the Supplementary Fig. S3.

It is known that AIM2 regulates AKT signaling in colorectal cancer^14,15,21^, and that AKT plays a vital role in modulating cancer cell proliferation and migration^22–24^. Hence, we asked whether the AKT signaling pathway plays a role in AIM2 regulation of GC cell proliferation and migration. In fact, silencing AIM2 led to a significant increase in the expression level of phosphorylated AKT (p-AKT) in both AGS and MKN45 cells (*P* < 0.01), but not in total AKT levels (Fig. 4a,b). Conversely, AIM2 overexpression had the opposite effect with regards to AKT phosphorylation (*P* < 0.01, Fig. 4c). Next, we treated AIM2-depleted cells with Ly294002 (an indirect inhibitor of AKT) and MK2206 (an AKT-selective inhibitor). Results from Western blots demonstrated that Ly294002 or MK2206 greatly suppressed activation of AKT within AIM2-deficient AGS and MKN45 cells (*P* < 0.05, Fig. 4a,b). Furthermore, our results showed that AIM2 knockdown in AGS cells inbibited the caspase-1 protein expression while AIM2 overexpression did the opposite in SGC7901 cells (Fig. 4d). In addition, colony formation assays (*P* < 0.001, Fig. 4e) and migration assays (*P* < 0.05, Fig. 4f) demonstrated that the enhanced proliferation and migration ability induced by AIM2 knockdown was partially impaired upon treatment with Ly294002. These results suggest that AIM2 suppresses GC cell proliferation and migration in an AKT-dependent manner.

## Discussion

As an intracellular DNA receptor, AIM2 determined the presence of dsDNA within the cytosol and binds ASC and caspase‐1 in order to form inflammasomes, which leads to the production of caspase‐1 and inflammatory factor IL‐1β. Currently, AIM2 is recognized to be both a tumor suppressor and an oncogene across different types of cancers. AIM2 expression is reported to be frequently down-regulated in several malignancies, including breast cancer^10^, hepatocellular carcinoma^11,12^ and colorectal cancer^13–16^. Additionally, loss of AIM2 expression is associated with oncogenic properties in these cancers. On the other hand, AIM2 is considered to be an oncogene in oral squamous cell carcinoma^17^, as well as non-small cell lung cancer^18,19^.

To date, there is only one study that has focused on AIM2 in GC, which demonstrates that AIM2 levels are lower in early GC tissues compared to progressive GC tissues and AIM2 overexpression suppresses GC cell proliferation, migration and invasion^20^. However, the clinical significance and the concrete molecular mechanisms behind the role of AIM2 in GC remain unclear and need to be further explored.

Herein, we determined that the expression of AIM2 in GC tissues is lower compared to the adjacent normal tissues, and is further reduced in patients with LNM, late TNM stage and a larger tumor size. Moreover, clinical analyses have also revealed that AIM2 expression is significantly correlated to tumor size, LNM and TNM stage. Several studies^25,26^ have demonstrated that a lack of AIM2 expression may be utilized as a biomarker for identifying colorectal cancer patients with poor prognosis. Herein, we provide first evidence that GC patients with low AIM2 expression have a lower survival rate compared to those with high AIM2 levels. Importantly, further multivariate analysis recognized AIM2 as an independent prognostic factor for survival of GC patients. In addition, our loss-of-function and gain-of-function experiments suggest that AIM2 inhibits GC cell proliferation and migration, which suggests that AIM2 plays a tumor-suppressive role in GC.

AKT, a master regulator of cell survival, is often overexpressed and hyperactivated in various human cancers, including GC^22,27,28^. Considering that AIM2 regulates the AKT signaling pathway in colorectal cancer^14,15,21^, we hypothesized that AKT is likely implicated in AIM2-mediated inhibition of GC cell proliferation and migration. We discovered that knockdown of AIM2 led to enhanced phosphorylation of AKT, while AIM2 overexpression had the opposite effect in GC cells. In addition, the enhanced proliferation and migration ability induced by AIM2 knockdown was partially impaired upon treatment with the AKT inhibitor, which suggests that AIM2 inhibition of GC cell proliferation and migration is likely dependent on AKT.

In resting cells, AIM2 physically interacts with and limits the activation of DNA-dependent protein kinase (DNA-PK), which is a PI3K-related family member that promotes AKT phosphorylation^14^. Thus, in GC cells, loss of AIM2 might enhance DNA-PK-mediated AKT hyperactivation, thereby promoting proliferation and migration of GC cells. Conversely, AIM2-mediated suppression of GC cell proliferation and migration may be ascribed to inflammation-related factors, which also have a significant function in the initiation and progression of GC^29,30^. Researchers have suggested that AIM2 is able to activate inflammasomes, and act as a tumor suppressor by inducing IL-18 and IL-1β in GC cells^20^. In the present study, we confirmed that AIM2 promoted the caspase-1 protein expression in GC cells. Therefore, the suppressive effects of AIM2 in GC cell proliferation and migration might partially depend on inflammasome activation.

In conclusion, our experiments not only indicate the clinical significance of AIM2 in GC, but also confirm its inhibitory role in the regulation of GC cell proliferation and migration. Moreover, we reveal a possible novel mechanism for AIM2 inhibition of GC cell proliferation and migration, partially via its participation in suppressing the AKT signaling pathway. Our findings demonstrate that AIM2 is an independent prognostic marker, as well as a potential therapeutic target, for treatment of GC.

## Materials and methods

### Tissue samples and cell lines

Human GC samples, as well as their corresponding adjacent normal tissues, were acquired from patients who underwent surgical resection without undergoing preoperative chemotherapy at the First Affiliated Hospital of Wannan Medical College from 2012 to 2013. The clinicopathological characteristics of these patients are shown in Table 1. The study protocols conformed to the standards of Declaration of Helsinki, and was granted approval by the Medical Ethics Committee of the First Affiliated Hospital of Wannan Medical College. Written informed consent was acquired from all patients.

The human GC cell lines (MGC803, SGC7901, MKN45 and AGS) were purchased from the Cell Bank of the Chinese Academy of Sciences (Shanghai, China), and cultured in the RPMI 1640 medium (Invitrogen, USA) containing 10% fetal bovine serum (FBS; Gibco, USA). Lentiviral vectors plasmids were constructed at GENECHEM Biotech at Shanghai, China. Stable GC cell lines were constructed in order to knockout and upregulate AIM2 expression using lentivirus (GENECHEM Biotech, Shanghai, China). Lentiviral transfection procedures were carried out as per the manufacturer’s instructions. The sequence specific for human AIM2 include 5′-CCCGAAGATCAACACGCTTCA-3′. GC cells were treated with Ly294002 (#S1737) or MK2206 (#SF2712) for 24 h, which were purchased from Beyotime (Beijing, China).

### Immunohistochemistry (IHC)

IHC staining of AIM2 was conducted according to the manufacturer’s instructions through the use of a primary antibody recognizing human AIM2 (dilution 1:200; #ab93015, Abcam). Staining intensity was classified as either 0 (no staining), 1 (weak staining), 2 (moderate staining) or 3 (strong staining). The staining percentage was designated as 1 (< 25%), 2 (25–50%), 3 (51–75%), or 4 (> 75%). The final staining score was calculated using the multiple of color intensity and positive cell percentage, which ranged from 0 to 12. Patients were classified into two groups, those with scores between 0–4 were considered to be none or low, while 5–12 were considered to be high.

### Protein extraction and western blot analysis

Proteins were isolated through the use of RIPA lysis buffer (Beyotime, Beijing, China) supplemented with protease inhibitors (Roche, CA, USA), as per the manufacturer’s protocol. Total proteins were separated using SDS-PAGE and transferred onto PVDF membranes, which were placed in blocking buffer with TBS-T buffer containing 5% non-fat milk, and incubated with specific primary antibodies overnight at 4 °C. After washing the membrane three times, it was then incubated with horseradish peroxidase-conjugated secondary antibodies. The proteins were visualized using chemiluminescence and signals were quantified using the ImageJ software (version: 1.4.3). Detailed protocols were previously described^31^. Antibodies that were used in this study include: anti-AIM2 (dilution 1:1000; #ab93015, Abcam), anti-p-AKT (Ser473) (dilution 1:1000; #4058, CST), anti-AKT (dilution 1:1000; #9272, CST), anti-caspase-1 (dilution 1:1000; #22915-1-AP, Proteintech) and anti-glyceraldehyde 3-phosphate dehydrogenase (GAPDH, dilution 1:5000; #AG019, Beyotime).

### RNA extraction and quantitative real-time PCR (q-PCR)

Total RNA was exacted from cell lines through the use of TRIzol reagent (Thermo Fisher Scientific), following manufacturer’s instructions. Next, we performed q-PCR on the ABI 7500 Fast Real-Time PCR System (Applied Biosystems, Waltham, MA, UK) with SYBR Green RT-PCR kits (ABI, USA). The human 18 s was used as endogenous control. The primer sequences included human-AIM2-Forward (5′-CAC CAA AAG TCT CTC CTC ATGTT-3′), human-AIM2-Reverse (5′-AAA CCC TTC TCT GAT AGA TTC CTG-3′), human-18 s-Forward (5′-GTA ACC CGT TGA ACC CCATT-3′), and human-18 s-Reverse (5′-CCA TCC AAT CGG TAG TAGCG-3′).

### Cell viability and migration assay

Cell viability was assessed utilizing a Cell Counting Kit-8 assay (CCK-8, APExBIO) according to the manufacturer’s instructions. The cell growth curve was plotted as a function of time and absorbance value (OD) at 450 nm.

For the colony formation assays, approximately 1000 cells were seeded onto 6-well plates for 10 days. Next, the colonies that were formed were washed with PBS, fixed with 4% paraformaldehyde (Beyotime) and stained using 0.1% crystal violet (Beyotime). Next, the images were captured using a digital camera (Nikon Corporation; Tokyo, Japan).

For transwell migration assays, GC cells within the serum-free medium were seeded into the upper chambers (Corning Incorporated; USA). The lower chamber contained medium with 10% FBS as a chemoattractant. After incubating for 12–48 h, the cells that had not migrated through the pores in the upper chambers were removed manually using a cotton swab. Cells that had migrated through the membrane were fixed in 4% paraformaldehyde (Beyotime), and stained using 0.1% crystal violet (Beyotime).

### Statistical analysis

Data is represented as mean ± standard error of the mean (SEM). A two-tailed Student t-test was used for comparison of variables of the two groups, and one-way analysis of variance (ANOVA) was utilized to compare multiple groups. Analysis of IHC results was carried out by the chi-square statistical test. Kaplan–Meier method with the log-rank test investigated the prognostic value of AIM2 for GC patients. Results with *P* < 0.05 were considered to be statistically significant.

## Supplementary Information

Supplementary Information
