# Supplementary material for: Absent in melanoma 2 suppresses gastric cancer cell proliferation and migration via inactivation of AKT signaling pathway
Source: Sci Rep. 2021 Apr 15;11:8235. doi: 10.1038/s41598-021-87744-4 (PMC8050218; doi:10.1038/s41598-021-87744-4)

Absent in melanoma 2 suppresses gastric cancer cell proliferation and migration via inactivation of AKT signaling pathway

Dong Wang[1], Junwei Zou[2], Jun Dai[1], Zhengwu Cheng[3,*]

[1] Department of Hepatobiliary Surgery, The First Affiliated Hospital of Wannan Medical College, Wuhu 241000, China.

[2] Department of General Surgery, The Second Affiliated Hospital of Wannan Medical College, Wuhu 241000, China.

[3] Department of Gastrointestinal Surgery, The First Affiliated Hospital of Wannan Medical College, Wuhu 241000, China.

[*]Correspondence to: Dr. Zhengwu Cheng, E-mail addresses: chengzhengwu1104@126.com. Department of Gastrointestinal Surgery, The First Affiliated Hospital of Wannan Medical College, No.2 Zheshan West Road, Jinghu District, Wuhu 241000, Anhui Province, China.

Supplementary Figure S1 Full scans of original blots for data in Figure 1.

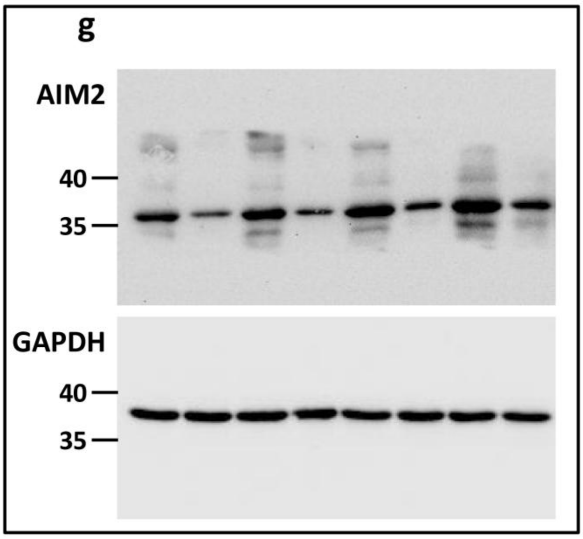

Supplementary Figure S2 Full scans of original blots for data in Figure 2.

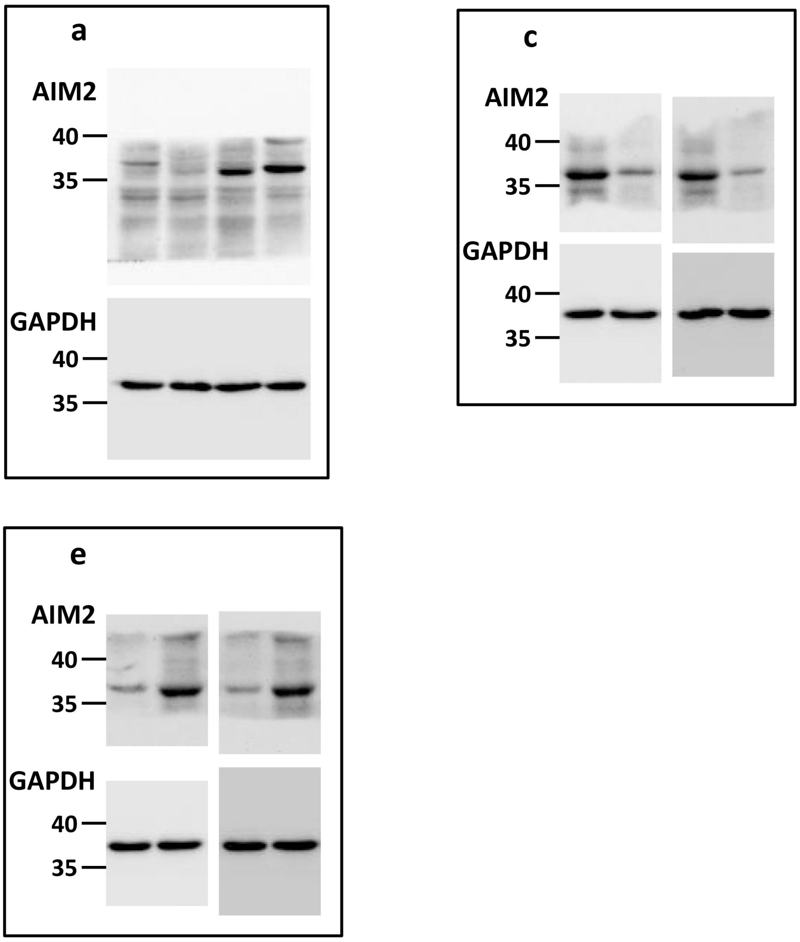

Supplementary Figure S2a

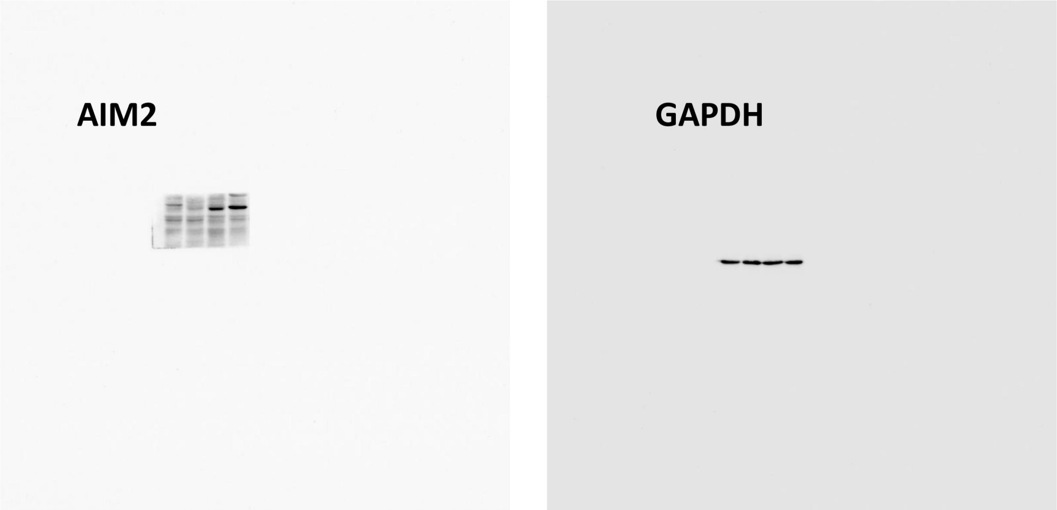

Supplementary Figure S2c

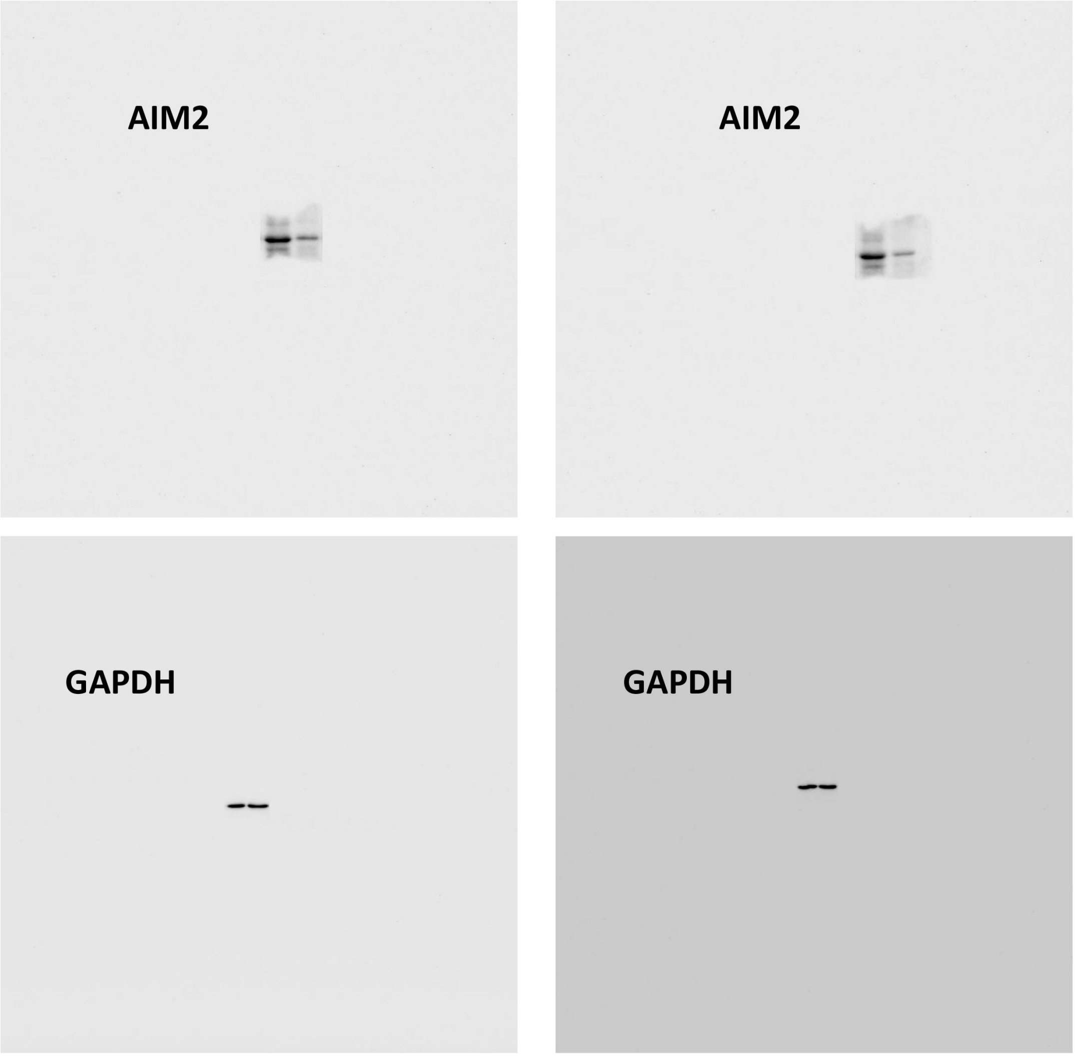

Supplementary Figure S2e

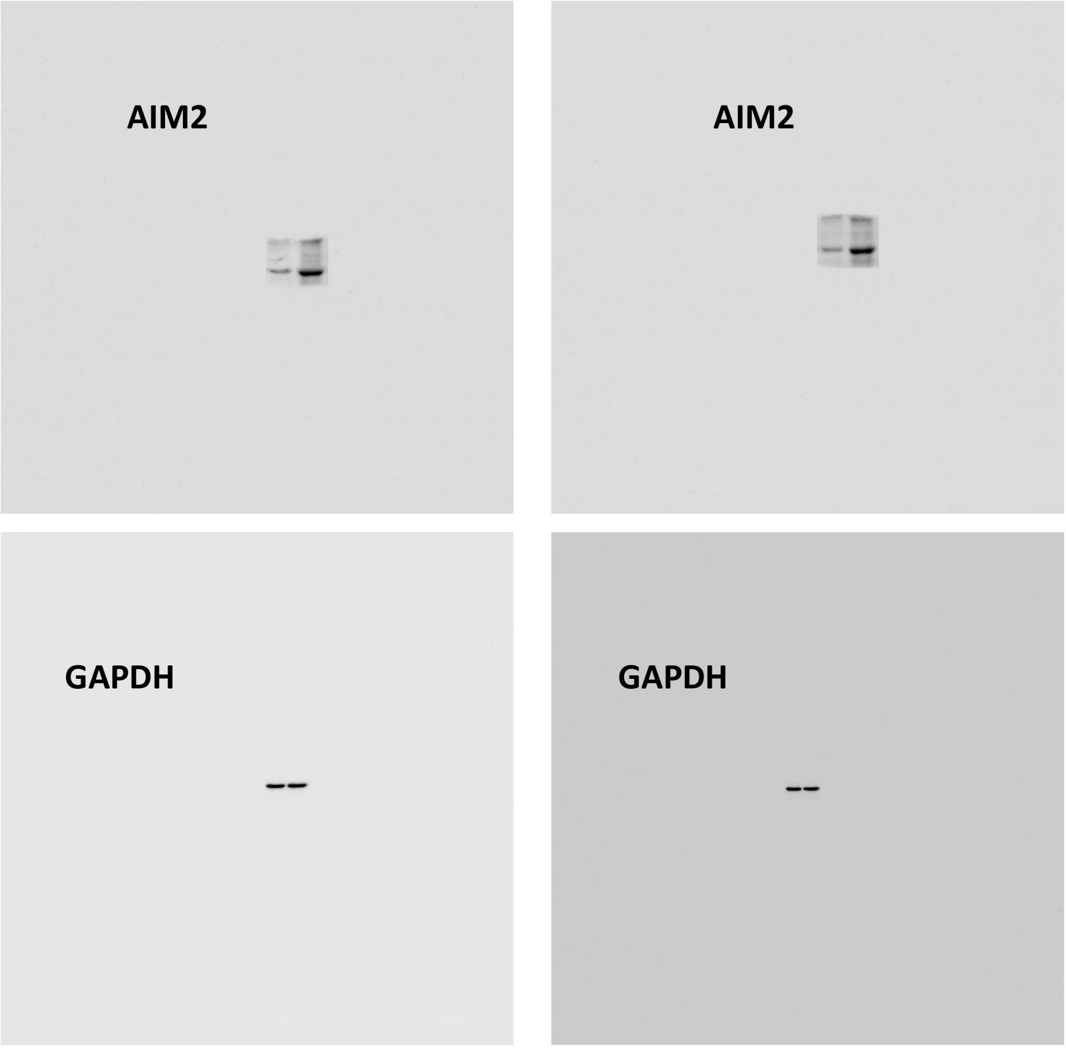

Supplementary Figure S3 Full scans of original blots for data in Figure 4.

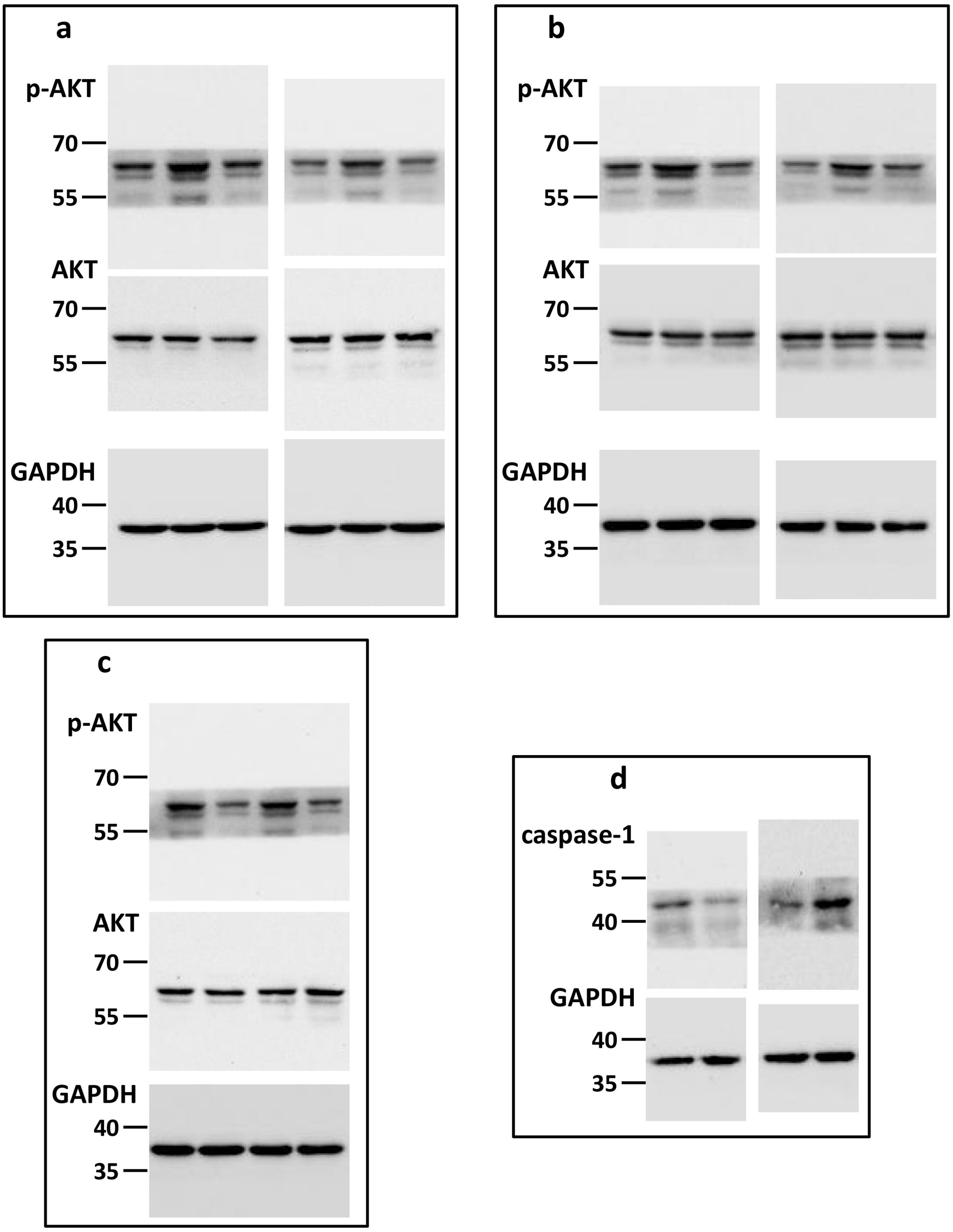

Supplementary Figure S3a

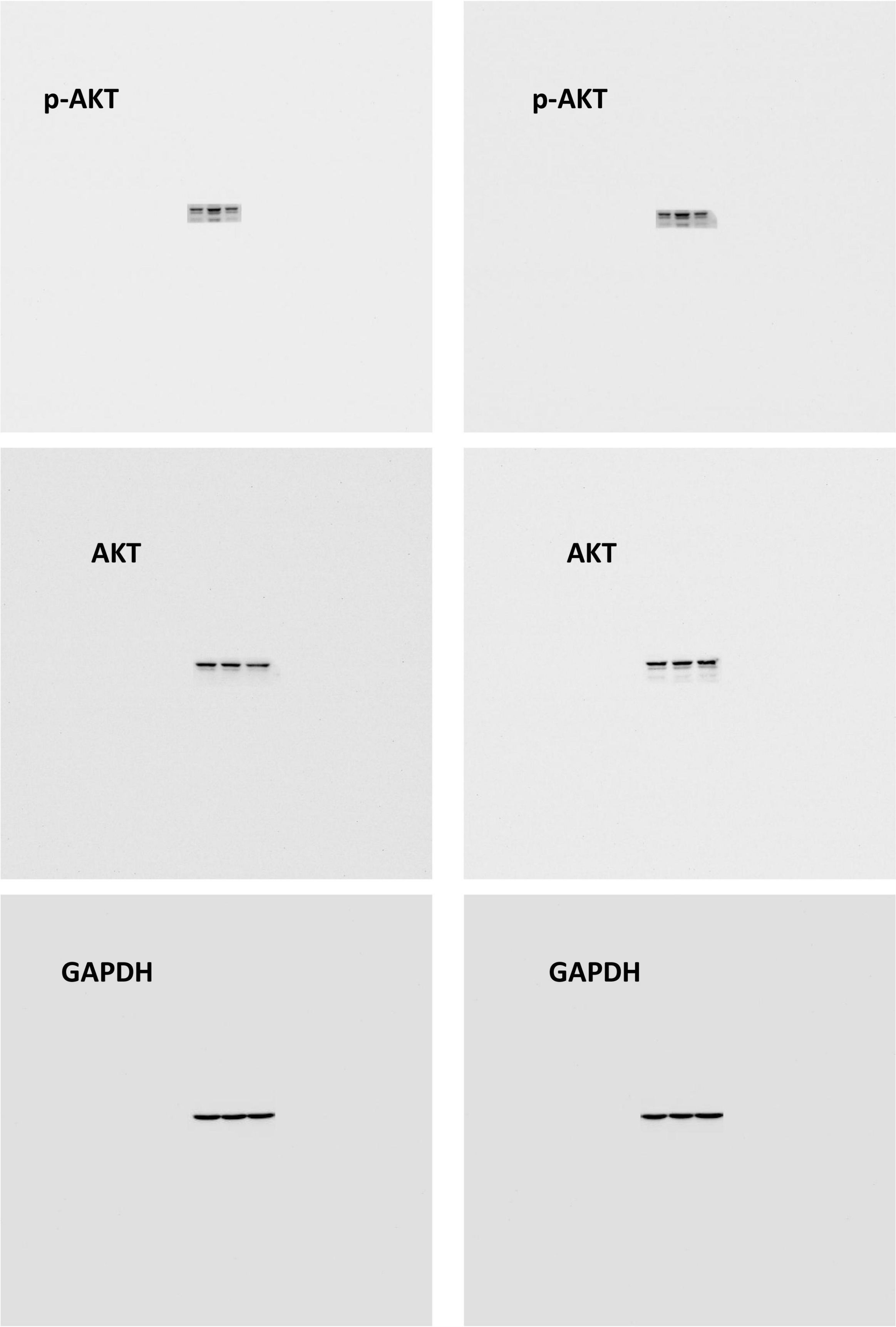

Supplementary Figure S3b

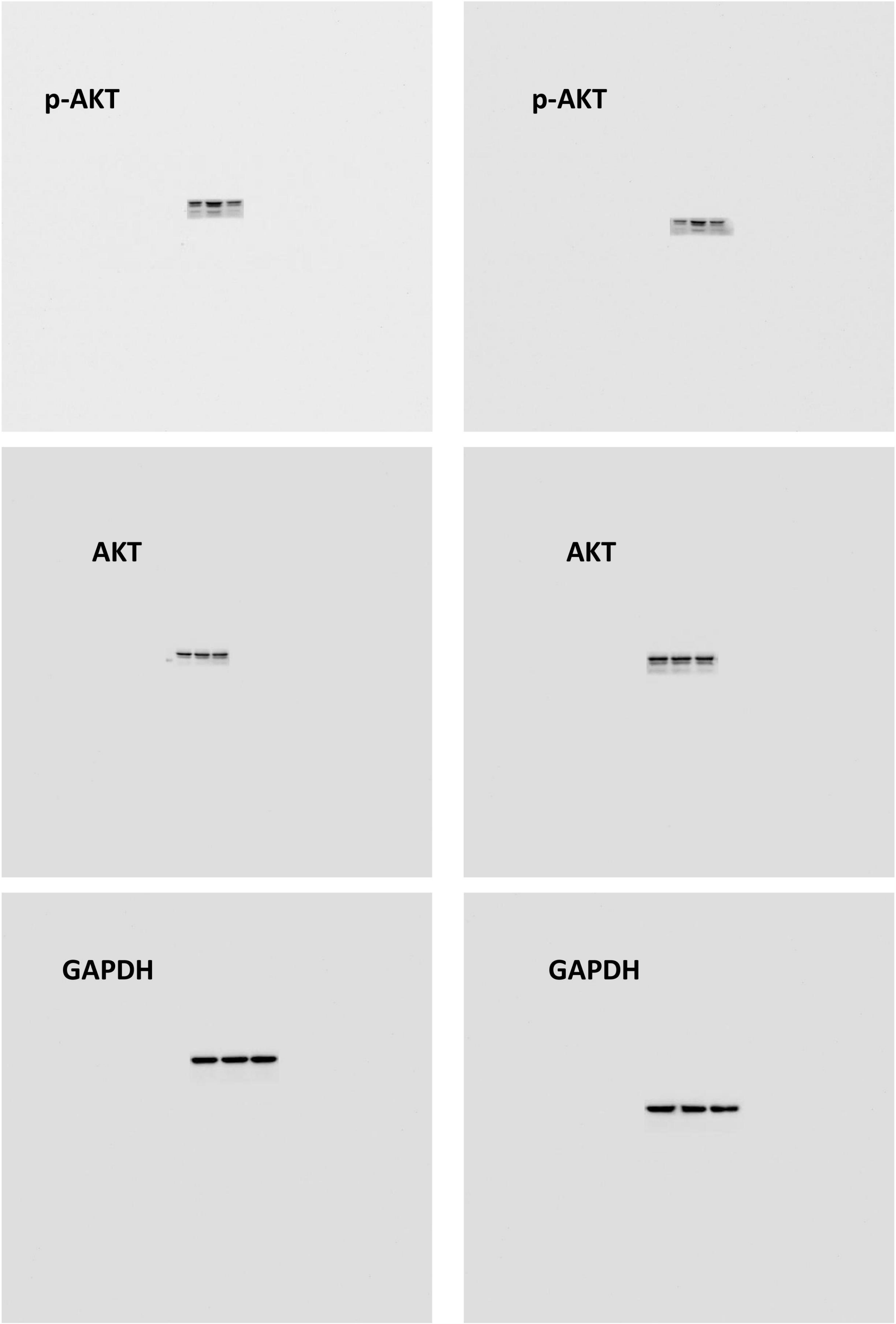

Supplementary Figure S3c

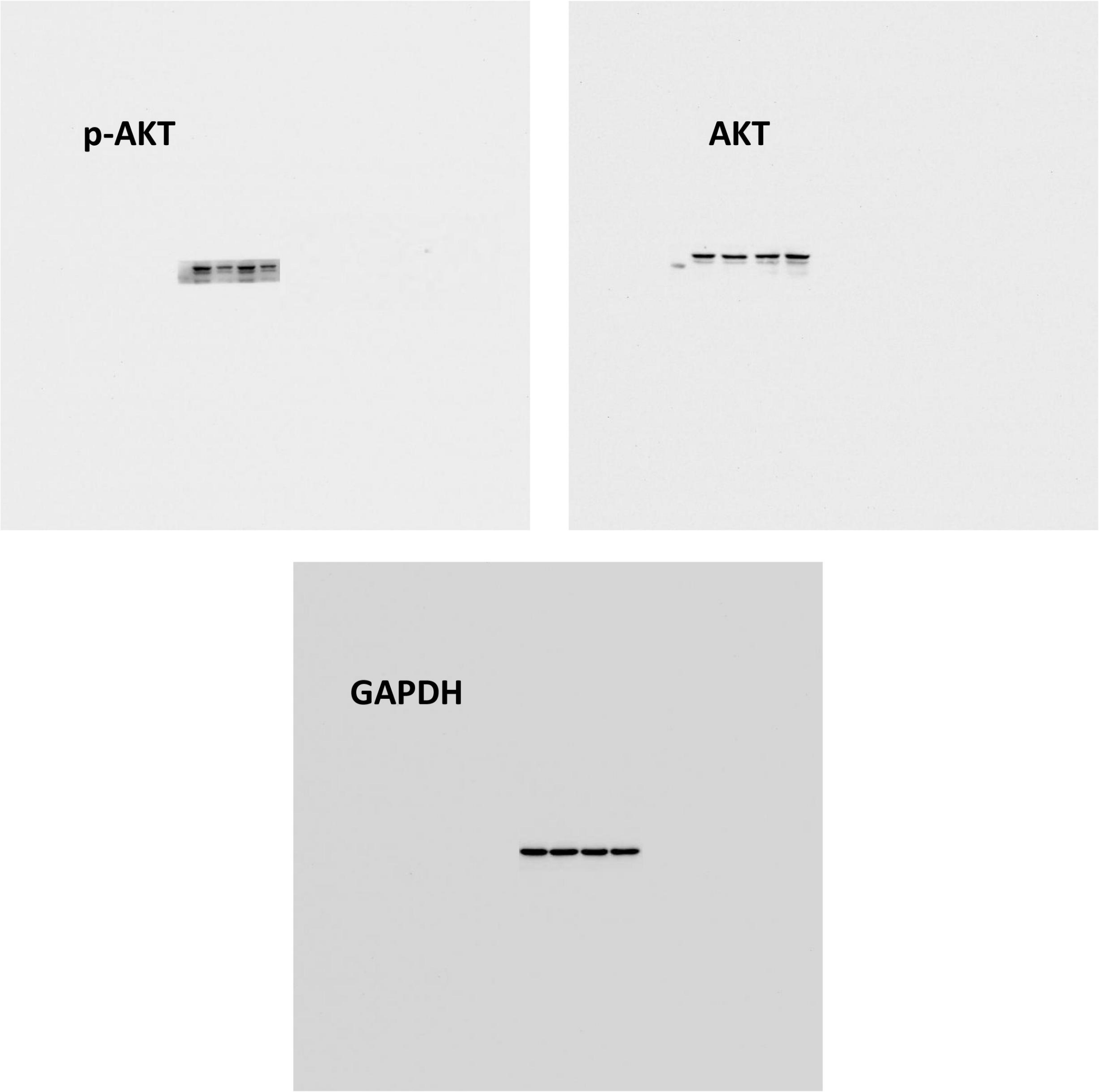

Supplementary Figure S3d

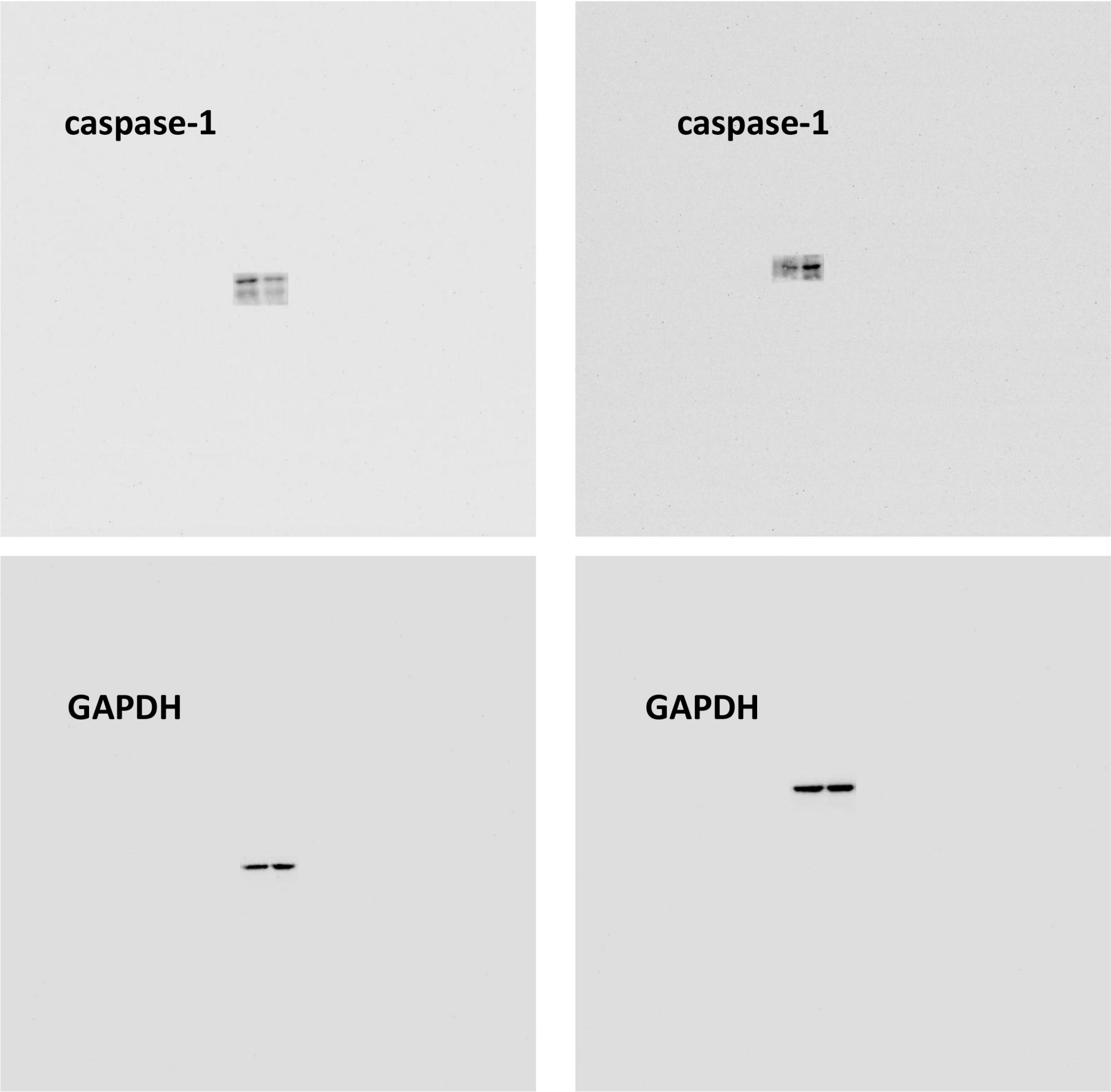

Supplement: Supplementary file 1 — Supplementary Information [file 41598_2021_87744_MOESM1_ESM.pdf]
